# Telomere Length and Mitochondrial Copy Number as Potential Biomarkers for Male Infertility in Iraqi Men

**DOI:** 10.3390/genes16121402

**Published:** 2025-11-24

**Authors:** Mustafa Faeq Kadhim, Farah Thamer Samawi, Ali Gargouri

**Affiliations:** 1 Faculty of Sciences of Sfax, University of Sfax, Sfax 3000, Tunisia; 2 Higher Institute for Infertility Diagnosis and Assisted Reproductive Technologies, Al-Nahrain University, Baghdad 10072, Iraq; 3 Higher Institute of Forensic Science, Al-Nahrain University, Baghdad 10072, Iraq; farahthamer1978@nahrainuniv.edu.iq; 4 Laboratory of Molecular Biotechnology of Euokaryotes, Centre of Biotechnology of Sfax, Sfax 3038, Tunisia

**Keywords:** infertile males, sperm, telomere length, mitochondrial DNA, copy number

## Abstract

**Background/Objectives**: Male infertility is one of the major problems in Iraqi health and society; it is caused by several factors, such as acquired, environmental, and genetic factors. Awareness of the crucial role of telomeres and mitochondria in sperm production and fertility has increased in recent years. This study aimed to evaluate the association between mitochondrial DNA (mtDNA) copy number and telomere length in sperm and the degree of infertility in Iraqi males. **Methods**: Of the 200 study participants, 50 were healthy controls and 150 were infertile. Sperm count, motility, and morphology were assessed by collecting and analyzing semen samples. After DNA extraction, the mitochondrial ND1 gene and the reference nuclear gene GAPDH were analyzed by quantitative PCR (qPCR) to determine the mtDNA copy number. To determine telomere length, another qPCR analysis was used. **Results**: The mtDNA copy number of infertile men was significantly higher than that of healthy controls with a *p*-value (0.001). In addition, the sperm of the patient group showed a significant reduction in telomere length (*p* = 0.001). According to the results of the study, male infertility in Iraqi men is associated with a higher number of mtDNA copies and shorter telomere length. DNA damage or a disruption in the mitochondrial energy production pathway could be the cause of this association. **Conclusions**: This study reveals that a higher number of mtDNA copies and shorter telomere lengths are associated with male infertility in Iraqi men. These results highlight the importance of continuing research and exploring new avenues in the field of male infertility.

## 1. Introduction

Infertility is a major social and psychological problem for couples in Iraq. It is defined as the inability to conceive after one year of unprotected sexual activity for women under 35 or six months for those over 35 [1]. Female factors account for about one third of infertility cases, followed by male factors for another third, and a combination of factors or unexplained reasons for the remainder [2]. Infertility can be attributed to a range of risk factors, including advanced age, smoking, alcoholism, obesity, malnutrition, and sexually transmitted infections. Global estimates of the prevalence of infertility range from 3.5% to 16.7% in developed countries and from 6.9% to 9.3% in underdeveloped countries [3,4].

In Iraq, infertility has a major effect on the sexual activity, self-image, and self-esteem of both men and women. Infertility has become much more common in Iraq over the past 16 years (2000–2016). The consequences of war, daily stress, smoking, work-related hazards, dietary habits, and genetic predispositions represent some of the causes associated with this trend [5].

While childbirth is often seen as a fundamental part of female identity, being a parent is seen by many Iraqi men as a sign of adulthood [5]. The high prevalence of infertility in Iraq is illustrated by the growth of IVF centers and infertility clinics in the country. Infertility in Iraq is poorly understood, despite its importance. The growing body of research on infertility serves as a bridge to fill the knowledge gap and improve our understanding of infertility and its negative impact on Iraqi society [6].

The World Health Organization (WHO) defines primary infertility as the inability of a couple to conceive despite regular, unprotected sexual intercourse. This condition only applies to individuals who have never been pregnant. Secondary infertility occurs when a couple who has previously conceived fails to conceive again within a year of unprotected sexual activity. More than 80 million people worldwide suffer from infertility, affecting 10–15% of couples. Secondary infertility is more common than primary infertility [7].

The amount of mitochondrial DNA (mtDNA) molecules present in a cell is indicated by its copy number. The “powerhouses of the cell,” or mitochondria, are organelles that produce energy. They contain DNA that is different from the nuclear DNA that exists in the nucleus of the cell. Mitochondria play a critical role in sperm health by producing the energy needed for sperm production and function. A reduced number of mtDNA copies can compromise this energy production, leading to impaired sperm motility, viability, and overall fertility [8,9]. Male fertility is strongly influenced by mitochondrial DNA copy number. Reduced energy production, oxidative stress, apoptosis, and poor sperm maturation are all consequences of increased copy number that can lead to infertility. A better understanding of the link between male infertility and mtDNA copy number could help in the creation of new diagnostic and therapeutic approaches [10].

Sperm mitochondrial DNA copy number (mtDNA-CN), a relative measure of mtDNA content, has also been reported to be negatively correlated with fertilization rate. Higher sperm mtDNA-CN is associated with lower pregnancy probability in couples without contraception, suggesting that mtDNA might be a potential clinical biomarker to predict male fecundity [11]. Studies reveal that telomeric DNA damage often precedes mitochondrial metabolic disorders, with patients suffering from mitochondrial diseases displaying shorter telomeres than healthy individuals [12]. Consequently, mitochondrial dysfunction has been linked to telomere attrition, and telomerase activation has been shown to reverse this decline [13]. Telomeres are DNA–protein complexes located at the ends of chromosomes and consist of a non-coding hexamer formed by the tandem formation of a highly conserved repetitive DNA sequence (TTAGGG), forming a T-loop structure that interacts with the Shelterin protein complex to form a fully functional hooded structure [14]. The specific structure allows cells to distinguish telomeres from sites of DNA damage, protects them from inappropriate DNA repair mechanisms, prevents gene degradation due to incomplete DNA replication, protects chromosome ends from erosion, and plays a crucial part in the integrity of the structure and stability of the chromosomal genome itself and preserving the essential biological functions of DNA [15]. More recent studies have suggested that STL may be a promising marker of male reproductive biology [16]. Most studies have concluded that STL is shorter in men with idiopathic infertility compared to fertile men. Telomere length is positively correlated with sperm anterograde motility and sperm count and negatively associated with sperm DNA fragmentation, and can be used as a marker of sperm quality [17]. However, some scholars have suggested that telomere length shortening may be a sign of sperm damage rather than a cause of sperm alteration [18]. The current study was conducted to evaluate mtDNA copy number and telomere length in infertile men, compared to a control group.

## 2. Materials and Methods

### 2.1. Subjects

This study included 200 semen samples from 150 infertile Iraqi men and 50 fertile Iraqi men (used as a control group). The infertile men were classified into three groups based on their semen parameters according to the World Health Organization (WHO) semen analysis criteria [19]:

- Group 1 (A): 49 men with asthenozoospermia;
- Group 2 (OA): 44 men with oligoasthenozoospermia;
- Group 3 (OAT): 57 men with oligoasthenoteratozoospermia.

### 2.2. Ethical Approval

Semen samples were collected from the High Institute for Infertility Diagnosis and Assisted Reproductive Technologies between April 2022 and February 2023. The study, conducted at Al-Nahrain University’s Forensic DNA Research and Training Center (reference no. 998, 13 February 2022), adhered to the ethical principles outlined in the Declaration of Helsinki. All participants provided informed consent before sample collection.

### 2.3. Semen Analysis

#### 2.3.1. Macroscopic Examination

Appearance: Typically opalescent or grayish-white. Abnormal colors, such as brown or red, may indicate the presence of blood. Volume: measured using a graduated test tube. The normal volume varies from 1.5 to 5.0 milliliters per ejaculation. Hypovolemia is diagnosed when the volume is less than 1.5 mL. pH: Measured using Litmus paper. The normal range is 7–8.0. Liquefaction: should occur within 30 min to 1 h at 37 °C or room temperature. Viscosity: assessed by observing the flow of semen through a pipette. Normal semen should form a single small drop and flow smoothly.

#### 2.3.2. Microscopic Examination

Sperm concentration: determined by counting sperm in a small sample under a microscope and multiplying by a factor to estimate the concentration per milliliter. The normal sperm count varies from 20 to 150 million sperm per milliliter. Sperm motility: evaluated by observing the movement of sperm under a microscope. Motility is categorized as progressive (PR), non-progressive (NP), or immotile (IM). Sperm morphology: assessed by examining the shape and structure of sperm under a microscope. Normal sperm morphology is characterized by a specific shape and size.

### 2.4. Sperm DNA Extraction, Purity, and Concentration

According to the manufacturer’s instructions, DNA is extracted from sperm using a spin column chromatography technique with the Presto™ Sperm DNA Extraction Kit (cat.no. GSP100), manufactured by Geneaid, Taiwan. By lysing the sperm cells and degrading proteins, the process allows the DNA to attach to the transparent fiber matrix of the spin column. A low-salt elution buffer is then used to elute the pure DNA after removing contaminants using wash buffers. No alcohol precipitation or phenol/chloroform extraction is required, and the entire process can be completed in just ten minutes. PCR and other enzymatic reactions can be performed with the purified DNA. DNA concentration and purity were assessed using a OneCNanodrop spectrophotometer (Thermo Fisher Scientific, Waltham, MA 02451, USA) [20]. DNA purity was determined by calculating the A260/A280 ratio, which should ideally be between 1.7 and 1.9. A ratio within this range indicates a pure DNA sample.

### 2.5. Quantification of Sperm mtDNA Copy Number by Real-Time PCR

#### 2.5.1. Principle

The most common methods to measure mtDNA copies are PCR-based, with qPCR being the most widely used due to its simplicity. Most protocols assess relative mtDNA copy number by comparing mtDNA levels to a nuclear gene, making comparisons between studies difficult. This study followed this procedure to ensure the accuracy of the results and obtain valuable feedback. Absolute mtDNA copy number can be determined using qPCR with a standard curve [21,22]. In the current study, the mitochondrial gene NADH dehydrogenase subunit 1 (ND1) served as the target for mtDNA quantification, while Glyceraldehyde-3-phosphate dehydrogenase (GAPDH) was used as the reference nuclear gene.

#### 2.5.2. Procedure

Relative mitochondrial DNA (mtDNA) content was quantified using real-time polymerase chain reaction (PCR) targeting the ND1 gene and normalized by simultaneously measuring the nuclear DNA (nDNA)-encoded GAPDH gene. qPCR was performed using a real-time quantitative PCR system (Qiagen, Hilden, Germany) and a specific primer set, shown in Table 1. A reaction mixture was prepared separately for the mtDNA ND1 gene and GAPDH with a final volume of 20 µL in different tubes. Each reaction contained 10 µL of TransStart^®^ Tip Green qPCRSuperMix (TransGen, biotech, Beijing, China, AQ141-01) containing the fluorescent dye Eva green, 1 µL of forward and reverse primers, and 4 µL of DNA sample (diluted with TBE buffer and measured by nanodrop at 260/280 nm wavelength) for both mtDNA and nDNA. A non-template control (NTC) was prepared containing all components of the master mix except template DNA. The thermal profile of the quantitative PCR used to estimate copy number is described as follows: 94 °C for 1 min, followed by 35 cycles (denaturation at 94 °C for 10 s, annealing at 58 °C for 20 s, and then extension at 72 °C for 1 min). A melting temperature range of 55 °C to 95 °C was adopted to perform the melting curve and to confirm that no primer dimers were found [23].

### 2.6. Quantification of Sperm Telomere Length (STL) Expression by qPCR

#### 2.6.1. Principles

A Human Telomere Length Quantification by qPCR Assay Kit (Cat.no. EQ022-01), manufactured by (ELK Biotechnology Company, Wuhan, China), was used. The single-copy gene (SCR for Single-Copy gene Reaction) primers are designed to specifically recognize and amplify a 78 bp region of human chromosome 11. Quantitative Polymerase Chain Reaction (qPCR) is a powerful technique for measuring the relative abundance of specific DNA sequences. This method was adapted to quantify sperm telomere length. Relative Quantification using the 2-ΔΔCT method was used. The 2-ΔΔCT method compares the cycle threshold (CT) values of a target gene (telomere repeat sequence) to a reference gene (single-copy gene, known as 36B4, large ribosomal subunit protein uL10), which encodes the acidic ribosomal phosphoprotein P0 (RPLP0). Table 1 shows the primer sequence for the SCR and TP genes. The difference between the CT values (ΔCT) represents the relative abundance of the target gene normalized to the reference gene. A larger ΔCT indicates a lower abundance of the target gene. Finally, accurate ΔΔCT calculations require approximately equal amplification efficiencies for the target and reference genes. This must be validated for each specific study [24].

#### 2.6.2. Procedure and Reaction Components

The EnTurbo™ SYBR Green PCR SuperMix provides specific and sensitive detection over a wide range of DNA concentrations. The SYBR Green I dye binds to double-stranded DNA, allowing the non-specific detection of amplified products, while the Hot-Start Taq Polymerase prevents extension of primers binding to template sequences with low homology (mispriming) as well as the extension of primers binding to each other (primer–dimer formation) during reaction setup. The PCR is optimized for low-concentration templates to ensure accurate quantification. For each sample, two separate 20 µL PCRs were set up to independently amplify the single-copy gene and the telomere repeat (TP) regions. The specific reagents used for these reactions included (0.4 µL) primer stock solution (Telomere or SCR), 4 µL Genomic DNA Template, Reaction mix (10 µL), and (5.6 µL) RNase-free ddH2O. Amplification was performed using a thermal cycler programmed with the temperature profile described as (initial denaturation at 95 °C for 1 min, 40 cycles (denaturation at 95 °C for 30 s, annealing at 55 °C for 30 s, and extension at 72 °C for 1 min) and the same melting temperature range (55 °C to 95 °C) for melt curve profiling.

The amplification efficiency of the qPCR primers for both telomere repeats and the single-copy reference gene was validated prior to applying the 2^−ΔΔCT^ method. Serial dilution standard curves were generated for both targets, and amplification efficiencies were calculated from the slope of each curve. The efficiencies for telomere and reference gene amplification were within the acceptable range, with less than a 5% difference between them, ensuring the accuracy and reliability of the telomere length quantification.

### 2.7. Statistical Analysis

The IBM Statistical Package for the Social Sciences (SPSS) V26 (version 26) and GraphPad Prism version 8 were used for descriptive and numerical statistics. Using qPCR to quantify the relative abundance of mtDNA by comparing the expression of the target gene (ND1) to a reference gene (GAPDH), the relative mtDNA content is determined. Telomere length results were analyzed according to the ∆CT and ∆∆CT equations, performed as in [25,26].

## 3. Results

A total of 200 semen samples were analyzed, from 150 infertile men (patient group) and 50 fertile men (control group). The infertile men were classified into three subgroups based on WHO semen analysis criteria: Group 1 (A, n = 49) with asthenozoospermia, Group 2 (OA, n = 44) with oligoasthenozoospermia, and Group 3 (OAT, n = 57) with oligoasthenoteratozoospermia. The 50 fertile men served as controls.

### 3.1. Age of the Study Groups

The mean age ± SE for the infertile male group was (33.047 ± 0.81), while in the control group it was (30.880 ± 1.43). The results showed that there was no significant difference in age between the two groups (*p* = 0.1).

### 3.2. Demographic Characteristics

Table 2 shows that, compared with the control group, a significantly higher percentage of patients had a family history of the disease (*p* < 0.001). The study results showed that there were notable variations between patient and control groups in a number of biological, behavioral, and demographic factors that contributed to infertility. We show that smoking, education level, length of infertility, and family history of fertility are important contributors to male infertility, seen in Table 2.

These results suggest that the onset of the disease could have a genetic or environmental influence. Although the patient group has a higher percentage of alcohol consumers, the difference is not statistically significant (*p* = 0.01). This demonstrates that alcohol consumption, although it may be a risk factor, may not be an important indicator of the disease. There is also no statistically significant difference in the prevalence of smoking between the two groups (*p* value = 0.2). As with alcohol consumption, it also appears that smoking is not a significant risk factor for this particular disorder.

The treatment protocol may influence the outcome of the disease, as shown by the significant difference in treatment rates (*p* < 0.001). Further research on the effectiveness of different treatment approaches is needed. The percentage of miscarriages in wives of patients was significantly higher (*p* < 0.001). This can be a sign of underlying reproductive health problems or other variables that influence the disease and miscarriage. Given the significant difference in education levels (*p* < 0.001), it is possible that a higher risk of the disease may be related to lower education levels. Several variables, such as economic status, access to health care, or lifestyle choices, could be responsible for infertility. Longer periods of infertility may be attributed to an increased risk of the disease or may be the result of increased complications of infertility, as indicated by the substantial difference in duration of infertility (*p* < 0.001).

According to Table 2, infertility may be the result of a confluence of environmental, lifestyle, and genetic variables. The condition appears to be significantly associated with factors such as family history, education level, and reproductive health problems. To clarify the underlying mechanisms and create efficient prevention and treatment strategies, further research is needed.

### 3.3. Seminal Fluid Parameters

In terms of sample volume and sperm count, the study found statistically significant differences between the patient group and the control group, seen in Table 3. The mean sample volume of the patient group was significantly smaller than that of the control group (3.11 mL vs. 4.59 mL, *p* < 0.001). The patient group also had a significantly reduced mean sperm count (15,947 vs. 39,800 millions/mL sperm, *p* < 0.001). These findings suggest a potential link between reduced sample volume and sperm count and the medical condition studied. Both of these variables were significantly lower in the patient group than in the control group. These results could have important therapeutic implications and need to be confirmed by further research.

Male infertility variables may contribute to the etiology of the disease, as evidenced by significant variations in sperm motility and morphology (*p* < 0.001).

### 3.4. mtDNA Copy Number and Telomere Length

The qPCR analysis using the 2^−ΔΔCt^ method revealed a noticeable reduction in relative telomere length among infertile males compared with the control group. The mean Ct for the telomere primers was 8.124 in the infertile group and 14.59 in controls, while the single-copy gene showed Ct values of 16.269 and 23.666, respectively. Accordingly, the ΔCt values were −8.145 for the infertile group and −9.076 for the controls. The resulting ΔΔCt was 0.931, yielding a relative telomere length (2^−ΔΔCt^) of 0.524 in the infertile group compared with 1.000 in the control group. These findings indicate significant telomere shortening in infertile men. The Ct values for GAPDH showed stable amplification across samples, ranging from 11.12 to 13.79 in the infertile male group and 11.12 to 13.36 in the control group, confirming its suitability as a reference gene for mitochondrial DNA copy number estimation. In contrast, ND1 displayed higher and more variable Ct values, with infertile males showing a broad range from 11.76 to 21.09, whereas controls showed a narrower and consistently lower range of 12.43 to 15.79. The qPCR amplification data and dissociation curve are provided in Figure S1 of the Supplementary File.

**Figure 1:**
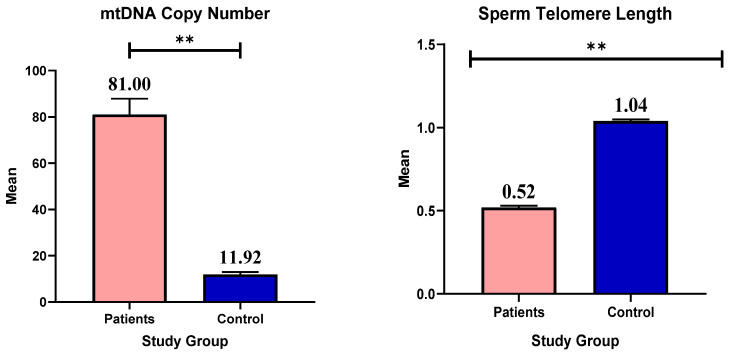
Mean and standard error of mtDNA copy number and telomere length in patients and control, **: statistically highly significant.

Telomere length and mtDNA copy number are two variables that are statistically compared between several patient groups and a control group in the data tables presented. The mean values of the two variables are compared in Figure 1 below and (Table S1 in the Supplementary File) between the control group and the patient group. The standard error of the mean is also shown, which helps to measure the precision of the mean. The difference between the two groups is statistically significant, as indicated by the *p*-value. The result showed that patients have a significantly higher mean copy number of mtDNA than controls (*p* < 0.001). In terms of telomere length, patients had a considerably lower mean telomere length than controls (*p* < 0.001).

**Figure 2:**
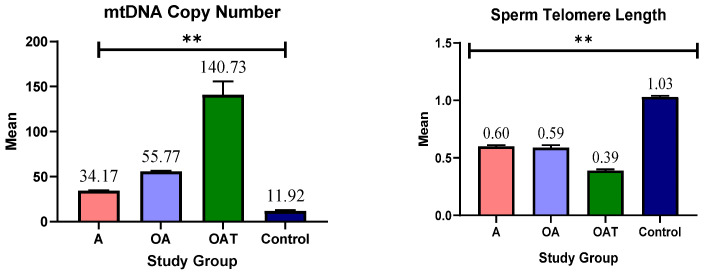
Comparisons of copy number and telomere length in patient subgroups and control group; **A:** Asthenozoospermia, **OA:** Oligoasthenozoospermia, **OAT:** Oligoasthenoteratozoospermia, ** statistically highly significant.

The qPCR result of the ND1 gene is shown as an example in Supplementary Figure S1. Supplementary Figure S2 shows the qRT-PCR result of telomere length. The mean values of mtDNA copy number and telomere length can be distinguished in Figure 2 (Supplementary Table S2) between the control group and various subgroups of patients. Significant differences between groups are indicated by the letters a, b, and c. Patients with asthenozoospermia have significantly lower copy numbers than those in the control, oligoasthenozoospermia, and oligoasthenoteratozoospermia. Compared with the oligoasthenoteratozoospermia and control groups, oligoasthenozoospermia patients have significantly reduced copy numbers. Patients with oligoasthenoteratozoospermia have significantly higher copy numbers than any other group. Telomere length is significantly reduced in patients with asthenozoospermia and oligoasthenozoospermia compared with the control group. Patients with oligoasthenoteratozoospermia express the lowest telomere length among all the groups. The results imply that, compared with healthy controls, patients with different levels of male infertility have notable changes in both copy number and telomere length. These results could be used as biomarkers for diagnosis and prognosis and may shed light on the underlying causes of male infertility.

Patients with oligoasthenoteratozoospermia had a significantly higher copy number than any other group, including the control group. This implies that this specific subgroup has abnormal gene copying machinery. Compared with all other groups, patients with asthenozoospermia showed a significantly lower copy number. Moreover, compared to both the oligoasthenoteratozoospermia and control groups, patients with oligoasthenozoospermia had a milder reduction. This suggests that within these two groups (oligoasthenoteratozoospermia and oligoasthenozoospermia), there is a mutation or defect in specific genes responsible for maintaining the number of mtDNA copies within a normal range. Regardless of all patient groups, the control group had the lowest copy number value, indicating that changes in copy number could indicate fertility complications.

Overall, the mean telomere length of the patient subgroups was significantly shorter than that of the control group, which included healthy individuals. Telomere destruction, which is often related to aging and cellular damage, is indicated by this shortening in individuals. All patient groups showed an overall decrease in telomere length, but these groups differed statistically significantly from each other. Compared with all other groups, including asthenozoospermia and oligoasthenoteratozoospermia, the oligoasthenoteratozoospermia group had the shortest mean telomere length. This implies that the reproductive cells in this group have more cellular damage. Finally, the telomere length of the asthenozoospermia and oligoasthenozoospermia groups was significantly shorter than that of the control group, suggesting that these groups had cellular DNA damage.

### 3.5. Correlation Test Between mtDNA Copy Number and Telomere Length

According to the current study, there is a moderate negative correlation between the telomere length and mtDNA copy number in all study groups, including patient subgroups and the control group. Both variables have a negative association, as indicated by the value of the correlation coefficient (r = −0.5565), which means that telomere length decreases when mtDNA copy number increases and vice versa. A reverse connection is represented by a negative value, while a strong negative association is indicated by a number around −1.

**Figure 3:**
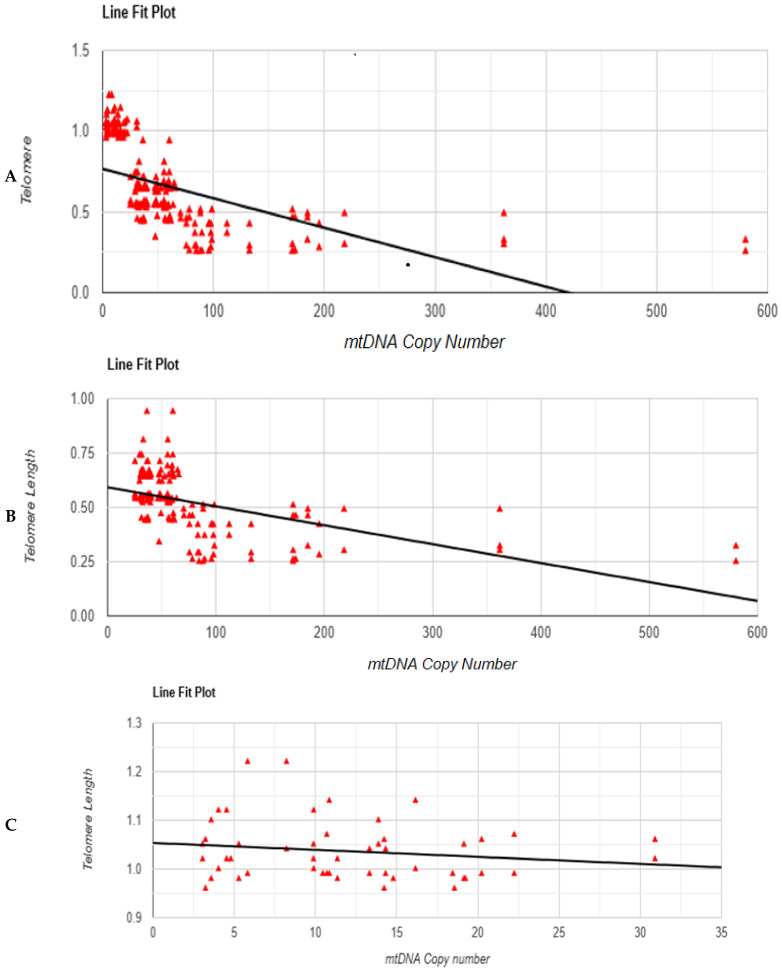
(**A**). Correlation test between mtDNA copy number mean and telomere length mean in all study groups, including patient subgroups and control. (**B**). Correlation test between mtDNA copy number and telomere length in patient subgroups (asthenozoospermia, oligoasthenozoospermia, and oligoasthenoteratozoospermia) with r^2^ = 0.25 and *p*-value < 0.01. (**C**). Correlation test between mtDNA copy number and telomere length in control group with r^2^ = 0.26 and *p*-value = 0.2 NS.

According to the coefficient of determination value (r^2^ = 0.3097), mtDNA copy number variations account for 30.97% of the telomere length variation. This demonstrates that telomere length is modulated by several factors other than the mtDNA copy number. The *p*-value (0.001) indicates a statistically significant correlation between the variables, indicating a substantial negative association between mtDNA copy number and telomere length. Figure 3A illustrates the result. Supplementary Figures S3–S5 show the correlation tests between the different subgroups of patients with controls (asthenozoospermia and control), (oligoasthenozoospermia and control), and (oligoasthenoteratozoospermia and control).

Correlation analysis between mtDNA copy number and STL (sperm telomere length) in patients with asthenozoospermia, oligoasthenozoospermia, and oligoasthenoteratozoospermia revealed an r^2^ value of 0.25 (r = −0.502), indicating that 25% of the variation in telomere length can be explained by changes in mtDNA copy number. However, the *p*-value ˂ 0.01 **, suggesting that this correlation is statistically significant. This implies that although there is a weak to moderate relationship between these two variables, the association lacks strong statistical support, and the observed correlation may be influenced by other factors or sample variability (Figure 3B).

Correlation analysis between mtDNA copy number and STL in the control group (Figure 3C) revealed an r^2^ value of 0.26, indicating that 26% of the variation in telomere length can be explained by changes in mtDNA copy number. However, the *p*-value was 0.2, which is not statistically significant (NS). This implies that even if there is a slight relationship, it is not strong enough to confirm a significant association, and other biological or methodological factors, as well as the increase in the number of control individuals, may influence the results.

### 3.6. Linear Regression Test Between mtDNA Copy Number and Telomere Length

Overall, a statistically significant linear association between mtDNA copy number and telomere length is provided by the regression model for all study groups (patients and control); the slope of the regression line is β = −0.0018, *p* < 0.001, as shown in Figure S6. This indicates that the expected telomere length decreases by an average of 0.0018 units for every unit increase in mtDNA copy number. As mentioned earlier in the correlation analysis, this negative coefficient denotes a negative association between the two variables. This coefficient is considered statistically significant if the *p*-value is less than 0.001. *p* < 0.001, α = 0.77: This represents the intercept of the regression line. When the mtDNA copy number is zero, this indicates the expected telomere length. However, since a copy number of zero may not be a realistic result, this interpretation may not be meaningful in the context of this particular study. The statistical significance of this intercept is indicated by the *p*-value < 0.001.

### 3.7. Receiver Operating Characteristic (ROC) Curve Analysis

The results of a Receiver Operating Characteristic (ROC) curve study are presented in Table 4. This study is frequently used to evaluate the performance of a diagnostic algorithm. In this case, the model uses two parameters: copy number and telomere length. The ability of the algorithm to distinguish between the two factors can be assessed by the area under the curve (AUC); values closer to 1 indicate greater accuracy. The threshold used to determine whether a sample is positive or negative is commonly referred to as the cutoff value. Specifically, specificity is the percentage of true negatives correctly identified, while sensitivity is the percentage of true positives correctly detected. The statistical significance of the results is illustrated by the *p*-value.

Overall, the results suggest that mtDNA copy number and telomere length are good predictors, with copy number performing slightly better in terms of specificity. The AUC value was 0.954 and 0.89 for copy number and telomere length, respectively, which is statistically significant (*p* < 0.001), while the cutoff value was 25.24 and 0.95, respectively. Table 4 and Figure S6 illustrated the results.

Assuming excellent and very good discriminatory power, respectively, mtDNA copy number and telomere length appear to be good predictors of disease. True positive and negative cases can be confidently identified by the model, based on the high sensitivity and specificity values for both parameters. It is essential to understand that the ideal cutoff point may change depending on the particular clinical context and the required ratio between specificity and sensitivity. Specificity should improve, but sensitivity will decrease with a greater cutoff point, and vice versa.

## 4. Discussion

The results of the current study showed no statistically significant differences in the average age of the study groups. However, demographic differences were significant with regard to the duration of infertility, education level, and family history. Semen parameters showed statistically significant differences in terms of sample size, sperm count, shape, and sperm motility, which were very low in patient samples when compared to the control group. Regarding the study aspect related to telomere length and mitochondrial DNA copy number, a significant increase in copy number was observed in the patient group compared to the control group. Furthermore, there were subset differences within the patient group, and telomere length was significantly lower in the patient group, particularly in the oligoasthenoteratozoospermia group.

The lack of significant differences between the average age of patients and healthy individuals suggests that age did not influence the results. However, family history, duration of infertility, and educational level were observed to differ significantly between the study groups, and these factors can increase the likelihood of fertility problems. This aligns with the findings of studies conducted by [27,28].

The results of the current study showed significant differences between the patient and control groups in terms of semen volume, sperm count, motility, and morphology. These findings are consistent with previous literature [29,30] linking decreased sperm concentration and count to increased oxidative stress, which is associated with mitochondrial dysfunction.

There was a significant increase in mitochondrial DNA copy number and telomere shortening in patients, in addition to clear differences between subgroups. These results can be explained by mitochondrial dysfunction or a compensatory response leading to this increase, while telomere shortening reflects DNA damage and increased oxidative stress. This is consistent with the results of Ling et al. (2023) [31] and Moustakli et al. (2023) [32]. This showed a negative relationship between telomere length and the number of mitochondrial DNA copies, confirming the role of mitochondria as a major source of oxidative stress, which in turn affects telomere length.

Both mtDNA-CN and STL are highly accurate diagnostic biomarkers, with mtDNA transcripts proving superior in differentiating between patient and healthy groups. This finding aligns with previous reports and studies indicating the potential of mtDNA-CN as an accurate diagnostic marker in male infertility [27,28], while STL can be used as an additional indicator to support diagnosis and help predict treatment outcomes.

The study results showed that an increase in the number of mitochondrial DNA copies is accompanied by a shortening of telomere length. The increased mtDNA copy number in patients suggests that mtDNA replication is dysregulated. The underlying reasons for this instability may be environmental, genetic, acquired, or a combination of all three. This could cause mitochondria to create excessive energy, which could lead to toxic reactive oxygen species that would seriously impact the sperm DNA and other components [33]. An elevated sperm mtDNA copy number is associated with damage to sperm and poorer measures of semen quality [34]. Mitochondria, the energy powerhouses of the cell, are essential for sperm health. An increase in mtDNA copy number can impair energy production, leading to decreased sperm motility, viability, and overall fertility [35]. Sperm mitochondrial function, encompassing energy production and apoptosis regulation, is vital during spermatogenesis; while the sperm mtDNA copy number is physiologically reduced during sperm maturation, elevated levels are associated with compromised semen quality, as shown in meta-analytic studies [34].

Mitochondria are essential for male fertility, playing a key role in germ cell development and sperm function. While ROS promote sperm capacitation, excessive levels induce oxidative stress, impairing sperm quality and motility. Thus, mitochondrial assessment is essential to understand male infertility [36]. Additionally, mitochondria generate reactive oxygen species (ROS), which can damage sperm DNA and other cellular components if not balanced with antioxidants. This oxidative stress can contribute to infertility [37].

The precise regulation of mtDNA copy number is fundamental to reproductive success; a high mtDNA copy number ensures the proper distribution of mitochondria, necessary for early embryonic development. Conversely, its downregulation is essential for optimal sperm function. Furthermore, an increase in mtDNA copy number can trigger apoptosis, or programmed cell death, leading to a decrease in sperm count and affecting overall fertility. Finally, mtDNA is involved in sperm maturation, and an increase in mtDNA copy number can disrupt this process, leading to abnormal sperm development and reduced fertility [10,36,38].

Male infertility can be caused by a variety of factors, including age, lifestyle, and environmental exposures, which can affect mtDNA copy number. Measuring mtDNA copy number may prove to be a useful biomarker for identifying men who would benefit from particular treatments and for detecting male infertility [39]. A previous investigation reported similar results to our study [40]. Their research examined mtDNA integrity and copy number in sperm from infertile men and found that individuals with oligoasthenoteratozoospermia (OATS) had a significantly higher mean mtDNA copy number compared to those with normal semen parameters who had asthenozoospermia. Specifically, they found that the mean mtDNA copy number was 15.7 in men with normal parameters, 34.3 with asthenozoospermia, 56.7 with oligozoospermia, and 73.7 with OATS [40]. Wu et al. conducted a study investigating the relationship between sperm mtDNA copy number, deletion rate, and infertility. Their findings suggest that a high mtDNA copy number in sperm is linked to male infertility, highlighting its potential as a sensitive biomarker to assess male reproductive health [41]. Similarly to the observations in our study, Jiang et al. (2017) show that increasing the mtDNA copy number can successfully reverse severe disease features resulting from mtDNA mutations, thus confirming the effectiveness of this strategy for treating mitochondrial disorders [42].

Telomere length has long been associated with aging. Telomeres act as protective caps on chromosomes, playing a crucial role in maintaining genomic integrity and cellular function. There is a strong link between aging and infertility in both sexes, with women often becoming infertile at an earlier age. In the last decade, telomeres have gained significant attention due to their role in fertility [43]. Telomeres, repetitive non-coding DNA sequences, play a critical role in chromatin integrity. Telomere length is age-dependent in somatic cells, but increases with age in sperm cells [44]. Sperm quality depends on various factors such as sperm count, motility, viability, reactive oxygen species (ROS) level, DNA fragmentation index (DFI), and telomere length. Sperm telomere length is critical during spermatogenesis, fertilization, pronucleus formation, and meiosis, although the exact mechanisms regulating sperm telomere length in male infertility are not fully understood. Sperm telomere length is directly associated with vitality, protamination, and progressive motility, and is negatively associated with DNA fragmentation. The link between sperm telomere length and its impact on male fertility is likely related to increased oxidative stress. Oxidative stress significantly damages hematopoietic stem cells and has been shown to be responsible for the dysfunction and aging of somatic and germ cells. Severe oxidative stress is one of the main factors responsible for male infertility and telomere shortening. Telomeres are rich in guanine residues, which are susceptible to oxidative stress, leading to increased sperm DNA damage, thereby reducing sperm quality and leading to infertility [45].

The results of the present study are consistent with those of Margiana et al. (2024), who showed that telomere length was decreased in an oligospermia group (*p*  <  0.0001) compared to a healthy group [46]. Similarly, the results are consistent with the study conducted by Dhillon et al. (2024), who assessed that the absolute length of sperm telomeres was significantly shorter (*p* = 0.004) in oligospermic individuals compared to the fertile group, and observed a significant positive correlation between absolute sperm telomere length and sperm parameters [47].

Similarly, Rajesh et al. (2021) showed that the mean (SE) of sperm telomere length (STL) recorded in infertility cases was significantly shorter than that of the control group: 140.60 (6.66) kb/genome and 239.63 (12.32) kb/genome, respectively (*p* < 0.001) [48]. A moderate positive correlation was evident between STL in kb/genome and the total sperm count in millions/mL (rho = 0.54, *p* < 0.001), progressive sperm motility (rho = 0.56, *p* = <0.001), and sperm viability (rho = 0.51, *p* = 0.032) in the infertile group [48]. Sperm telomere length can provide information about male fertility, as a shortened telomere can be a sign of impaired spermatogenesis, which can lead to a low sperm count, errors in chromosome segregation, and imbalanced gametes [49]. Moreover, the result of the current study was consistent with another study reported by Torra et al. (2018) [49], who showed that there were strong associations between sperm telomere length and sperm count; i.e., men with longer sperm telomeres tend to have a higher sperm count than those with shorter telomeres [50].

Furthermore, the results in the current study are consistent with those obtained by Darmishonnejad et al. (2020), who revealed that sperm telomere length was significantly shorter in infertile men than in fertile individuals, and observed significant associations between telomere length and sperm concentration, DNA fragmentation, and lipid peroxidation [44]. Furthermore, increased oxidative stress in sperm from infertile men may lead to abnormal chromatin packaging, DNA damage, and shorter sperm telomere length. Both mtDNA copy number and STL are indicators of cellular aging and have a comparable impact. Cellular function is compromised by telomere shortening and decreasing mtDNA copy number with cellular aging. Male fertility may be more significantly affected by the combination of short telomeres and decreased mtDNA copy number than by either condition alone [50]. A previous study, opposite to the current finding, found a consistent positive correlation between mtDNA copy number and telomere length in adults, suggesting a co-regulatory link between these factors, which may impact age-related and stress-related health problems [51].

The complex relationship between male infertility, telomere length, and mtDNA copy number requires further investigation. This includes investigating the fundamental processes that link these variables and identifying possible treatment targets to increase male fertility [52].

Male fertility is greatly impacted by the critical roles that telomeres and mitochondria play in cellular health and function. Several studies have demonstrated that sperm with reduced copies of mitochondrial DNA become less motile and viable, negatively impacting fertility. Telomeres and mtDNA have a reciprocal interaction in which each has a consequence on the other. Telomere shortening, for example, can be accelerated by the increased oxidative stress caused by an increase in mtDNA copies number [36,53].

Therefore, the combination of shortened telomeres and decreased mtDNA copy number affects mitochondrial function, reducing sperm energy production, causing DNA damage, and ultimately increasing the risk of infertility. Research in this area is essential to create new diagnostic tools to assess sperm quality and identify the fundamental causes of male infertility. It is also essential to create creative approaches to treat male infertility [32,35].

This study showed significant associations between increased mtDNA copy number, shorter telomere length, and male infertility; however, several limitations should be noted. Multivariate analysis was constrained because including certain clinical variables caused complete separation in the regression model, producing perfect but non-interpretable classification. Additionally, the sample size was relatively small, and significant demographic differences between groups, such as duration of infertility, education level, and family history, may have influenced the results. Environmental and lifestyle factors affecting oxidative stress were not fully assessed, and the cross-sectional design prevents establishing causality. Future studies with larger, balanced, and longitudinal cohorts are needed to validate these findings and clarify the independent effects of these genetic markers.

## 5. Conclusions

The results reveal that, compared to healthy controls, patients with varying degrees of male infertility exhibit significant differences in mtDNA copy number and telomere length. The elevated mtDNA copy number in the patient group suggests the dysregulation of mtDNA replication, which may be influenced by environmental or genetic factors. Male fertility and sperm telomere length were significantly associated, with shorter telomeres corresponding to reduced sperm quality and a higher likelihood of infertility. Studying the high mtDNA copy number in infertile Iraqi men could provide valuable diagnostic and prognostic indicators and help elucidate the underlying causes of male infertility. These findings contribute to reproductive health research and may support improvements in men’s health and psychological well-being.

## Figures and Tables

**Table 1:** The primer sequence used in the current study.

Primer	Sequence (5′→3′ Direction)	Size bp	Ref.
**ND1 for copy number using qPCR (58 °C)**			
Forward	ATTCGATGTTGAAGCCTGAGACT	108	[13]
Reverse	TGACCCTTGGCCATAATATGATT		
**GAPDH for copy number using qPCR (58 °C)**			
Forward	TGAGAAGTATGACAACAGCC	120	[14]
Reverse	TCCTTCCACGATACCAAAG		
**Telomere primer (55 °C)**			
Forward	CGGTTTGTTTGGGTTTGGGTTTGGGTTTGGGTTTGGGTT		[15]
Reverse	GGCTTGCCTTACCCTTACCCTTACCCTTACCCTTACCCT		
**Single-Copy gene Reaction (SCR) primers (55 °C)**			
Forward	CAGCAAGTGGGAAGGTGTAATCC		[15]
Reverse	CCCATTCTATCATCAACGGGTACAA		

**Table 2:** Demographic variables in patient and control groups.

Parameters		Group		Chi-Square	*p*-Value
		Patients	Control		
Family History	Yes	67 (44.67%)	1 (2%)	30.42	0.001 **
	No	83 (55.33%)	49 (98%)		
Alcohol	Yes	66 (44%)	6 (12%)	16.67	0.001 **
	No	84 (56%)	44 (88%)		
Smoking	Yes	48 (32%)	21 (42%)	1.66	0.2.
	No	102 (68%)	29 (58%)		
Treatment	Yes	102 (68%)	12 (24%)	29.62	0.001 **
	No	48 (32%)	38 (76%)		
Wife Miscarriage	Yes	25 (16.67%)	0	9.52	0.002
	No	125 (83.33%)	50 (100%)		
Education Level	No. Edu *	62 (41.33%)	7 (14%)	84.46	0.001 **
	P.S *	11 (7.33%)	0		
	S.S *	57 (38%)	2 (4%)		
	University	20 (13.33%)	41 (82%)		
Infertility Duration	1–5 Years	74 (49.33%)	0	200.00	0.001 **
	6–10 Years	32 (21.33%)	0		
	˃10 years	44 (29.33%)	0		
	No	0	50		

* No. Edu: No. education. P.S.: Primary school. S.S: Secondary school. **: statistically highly significant.

**Table 3:** Sample volume and sperm count in patient and control groups.

Parameters	Group	Mean	Std. Error Mean	*p*-Value
Volume (mL)	Patients	3.110	0.14	0.001 **
	Control	4.590	0.12	
Sperm Count (million/mL)	Patients	15.947	1.59	0.001 **
	Control	39.800	2.44	
	**Character**	**Patients**	**Control**	
Morphology of Sperm	Normal	100 (66.67%)	50	0.001 **
	Abnormal	50 (33.33%)	0	
Sperm Motility	PR	89 (59.33%)	50	0.001 **
	NP	30 (20%)	0	
	IM	31 (20.67%)	0	

PR: Progressive. NP: Non-progressive. IM: Immotile. **: statistically highly significant.

**Table 4:** Receiver operating characteristic curve (ROC).

Parameters	Area	Cutoff	Explanation	*p*-Value	Sensitivity%	Specificity%
mtDNA Copy Number	0.954	25.24	Excellent	0.001 **	95%	92%
Sperm Telomere Length	0.89	0.95	Very Good	0.001 **	95%	88%

**: statistically highly significant.

## Data Availability

The original contributions presented in this study are included in the article/Supplementary Material. Further inquiries can be directed to the corresponding authors.

## Supplementary Materials

The following supporting information can be downloaded at: https://www.mdpi.com/article/10.3390/genes16121402/s1, Table S1: Comparison in copy number and telomere length in patients and control group. Table S2: Comparison in copy number and telomere length in patients subgroups and control group. Figure S1: Quantitative Real-time PCR Analysis of ND1 gene: a. ND1 Amplification Plots qPCR amplification curves for samples from various research groups. CT values ranged from 11 to 19. b. Dissociation Curves: Melting temperature analysis of PCR products, ranging from 83 °C to 89 °C. Figure S2: qRT-PCR of Telomere length output for a sample of study group. Figure S3: Correlation test between mtDNA copy number and telomere length in Asthenozoospermia and Control with r2 = 0.72 and *p*-value ≥ 0.01. Figure S4: Correlation test between mtDNA copy number and telomere length in Oligoasthenozoospermia and Control with r2 = 0.83 and *p*-value ≥ 0.01. Figure S5: Correlation test between mtDNA copy number and telomere length in Oligoasthenoteratozoospermia and Control with r2 = 0.42 and *p*-value ≥ 0.01 **. Figure S6: Linear regression test between mtDNA copy number and telomere.
