# Supplementary material for: Telomere Length and Mitochondrial Copy Number as Potential Biomarkers for Male Infertility in Iraqi Men
Source: Genes (Basel). 2025 Nov 24;16(12):1402. doi: 10.3390/genes16121402 (PMC12733125; doi:10.3390/genes16121402)
Supplement: Supplementary file 1 [file genes-16-01402-s001.zip › genes-3993517-supplementary.pdf]

Table S1: Comparison in copy number and telomere length in patients and control group

Parameters	Group	Mean	Std. Error Mean	P-value
mtDNA Copy	Patients	81.00	6.85	0.001**
Number	Control	11.92	0.98	
Telomere	Patients	0.52	0.01	0.001**
Length	Control	1.04	0.01	

Table S2: Comparison in copy number and telomere length in patients subgroups and control group

Parameters	Subgroups	Mean	Std. Error	P-value
mtDNA copy number	Asthenozoospermia (A)	34.17^{ab}	0.60	0.0001**
	Oligoasthenozoospermia (OA)	55.77^{b}	0.79	
	Oligoasthenoteratozoospermia(OAT)	140.73^{c}	14.88	
	Control	11.92^{a}	0.98	
Telomere Length	Asthenozoospermia (A)	0.60^{b}	0.01	0.001**
	Oligoasthenozoospermia (OA)	0.59^{b}	0.02	
	Oligoasthenoteratozoospermia(OAT)	0.39^{a}	0.01	
	Control	1.03^{c}	0.01	

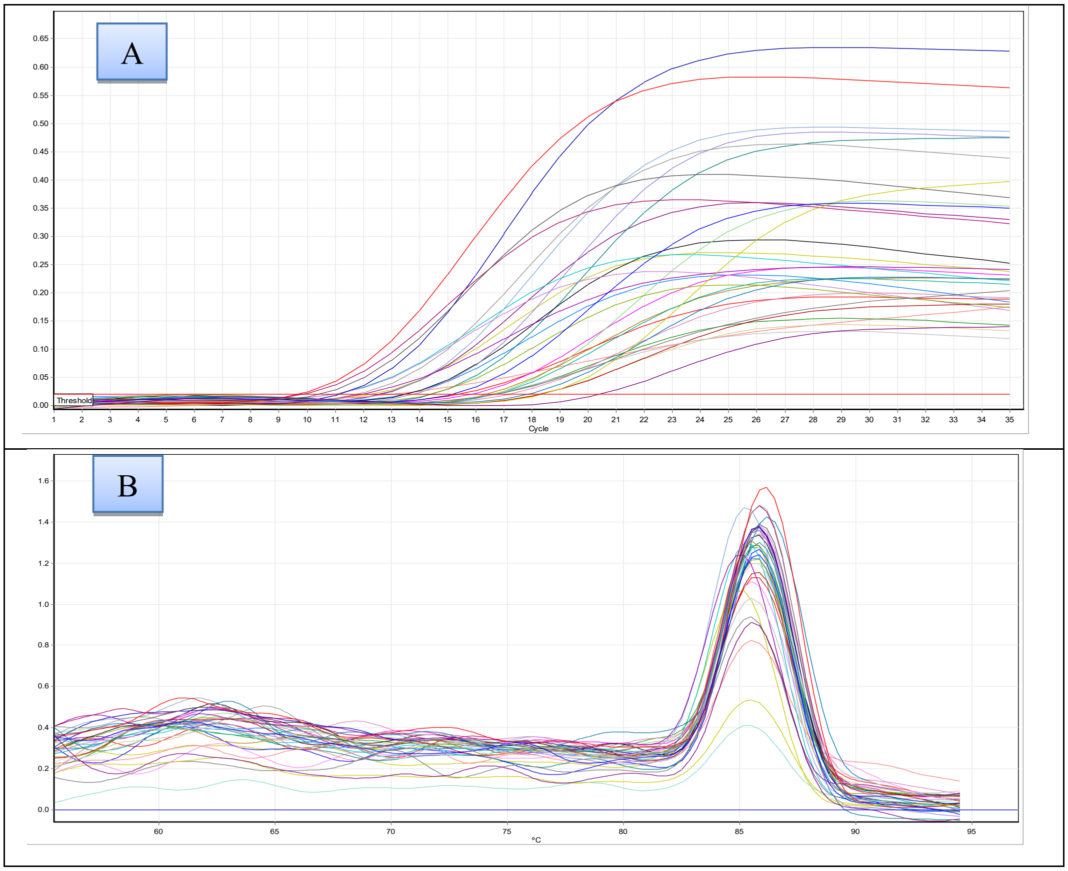

Figure S1: Quantitative Real-time PCR Analysis of ND1 gene: a. ND1 Amplification Plots qPCR amplification curves for samples from various research groups. CT values ranged from 11 to 19. b. Dissociation Curves: Melting temperature analysis of PCR products, ranging from 83°C to 89°C.

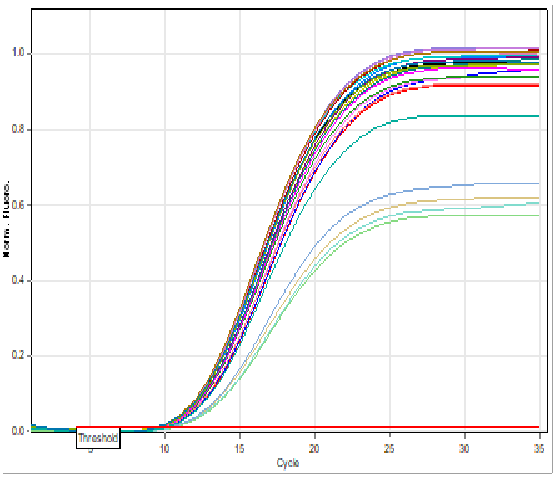

Patients sample (Telomere primer)

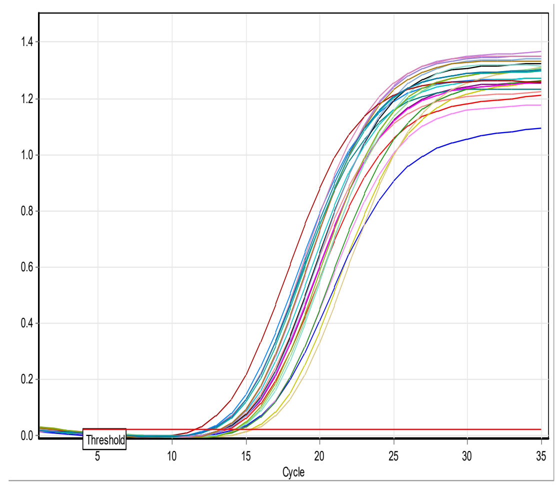

Control sample (Telomere primer)

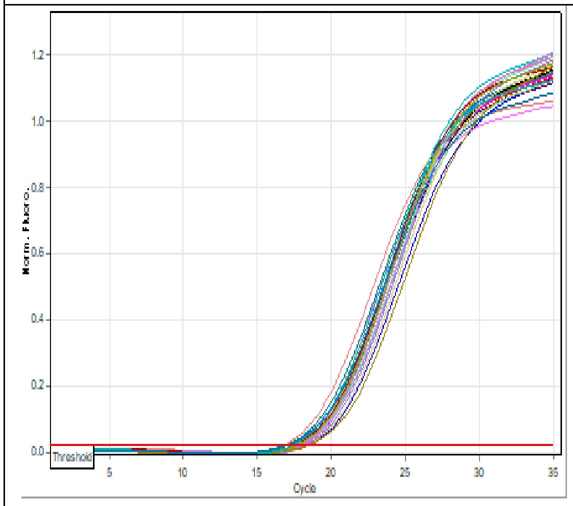

Patients sample (Single copy Gene)

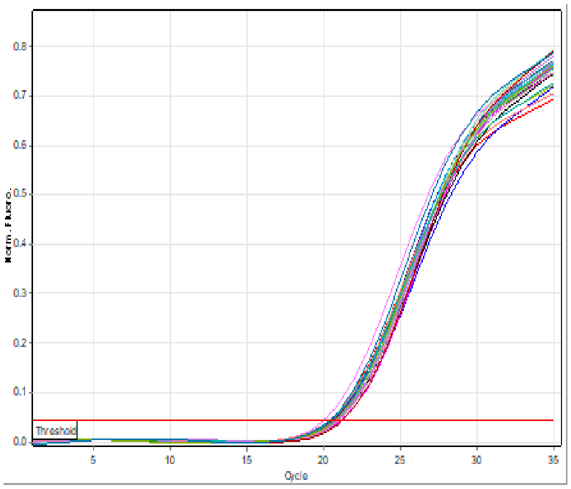

Control sample (Single copy Gene)

Figure S2: qRT-PCR of Telomere length output for a sample of study group

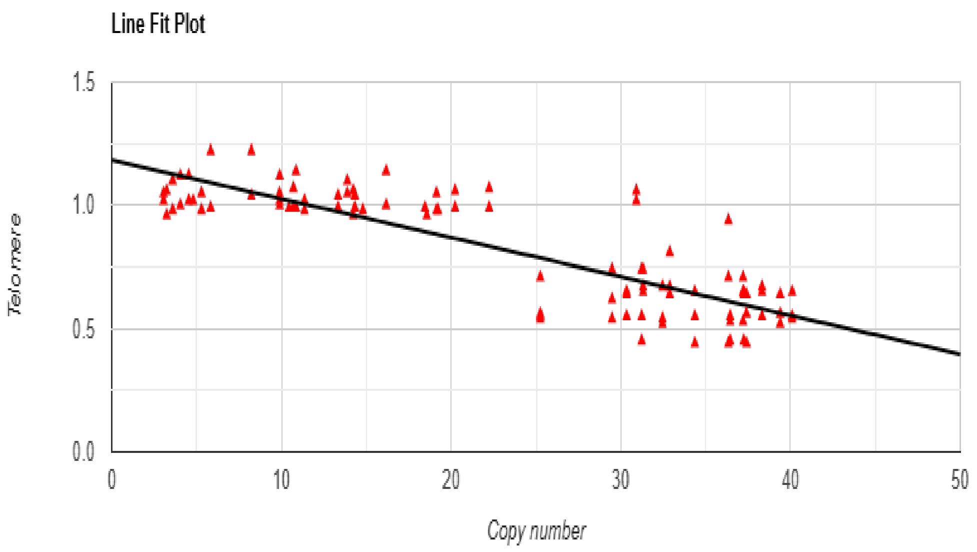

Figure S3: Correlation test between mtDNA copy number and telomere length in Asthenozoospermia & Control with r^2= 0.72 and *p*-value = >0.01**

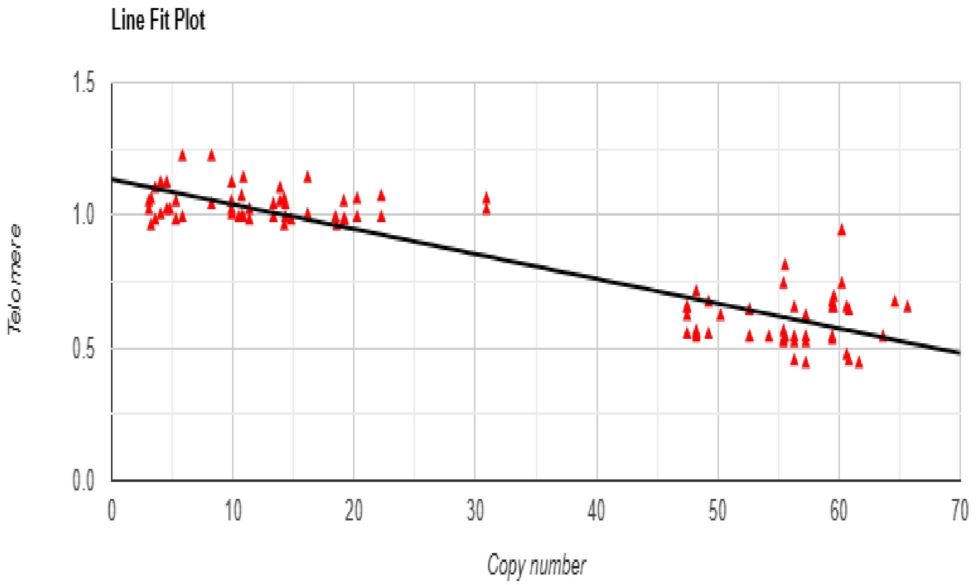

Figure S4: Correlation test between mtDNA copy number and telomere length in Oligoasthenozoospermia & Control with r^2= 0.83 and p-value = >0.01**

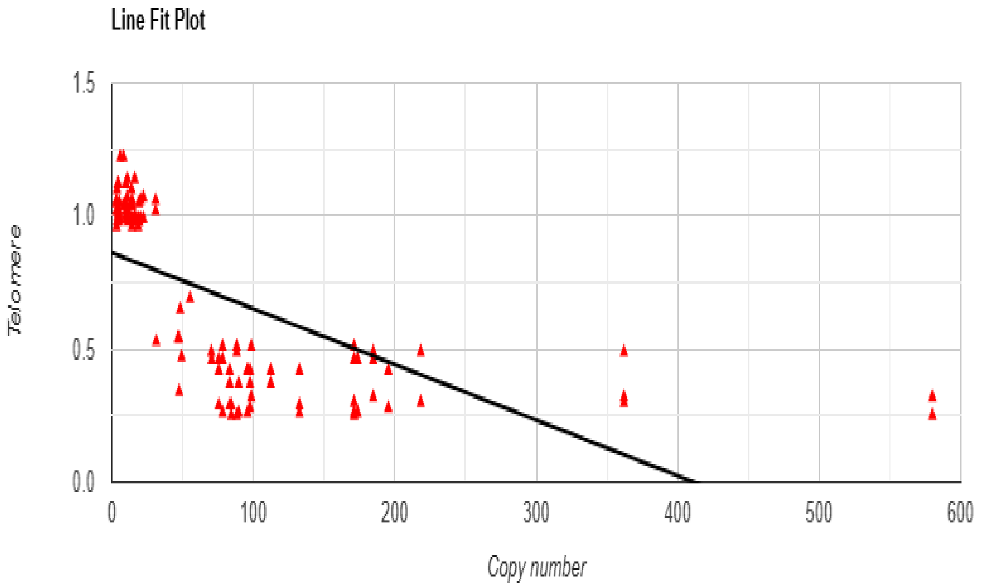

Figure S5: Correlation test between mtDNA copy number and telomere length in Oligoasthenoteratozoospermia & Control with r^2= 0.42 and p-value = >0.01**

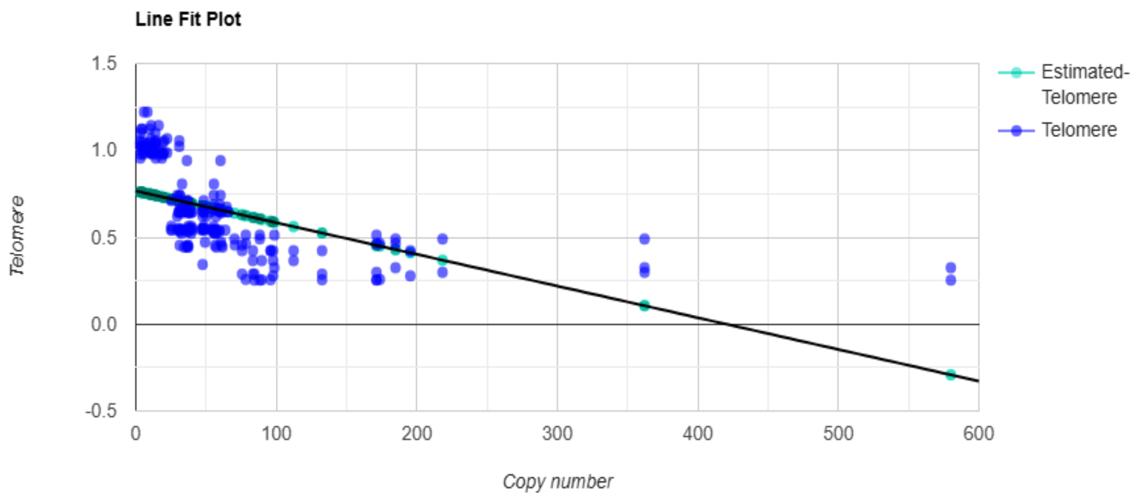

Figure S6: Linear regression test between mtDNA copy number and telomere ($\hat{Y}$ = 0.7663 - 0.001824X)
